# Cost-Effectiveness of Carotid Endarterectomy vs. Carotid Stenting: a Systematic Review and Meta-Analysis

**DOI:** 10.1007/s12975-025-01347-z

**Published:** 2025-04-11

**Authors:** Yash Akkara, Joshua J. Hon, Mahathir Ahmed, Basel Musmar, Joanna Roy, Stavropoula Tjoumakaris, Michael Reid Gooch, Robert H. Rosenwasser, Pascal Jabbour

**Affiliations:** 1 Imperial College School of Medicine, London, England; 2 Department of Neurological Surgery, Thomas Jefferson University Hospital, Sidney Kimmel Medical College, Philadelphia, PA USA

**Keywords:** Carotid Stenting, Carotid Endarterectomy, Cost, Cost-Effectiveness, Carotid Stenosis, Neurosurgery, Interventional Neuroradiology

## Abstract

**Introduction:**

Carotid artery stenting (CAS) and carotid endarterectomy (CEA) are gold-standard treatments of carotid artery stenosis. This study aims to identify the cost-effectiveness of CEA vs CAS.

**Methods:**

Studies were screened through PubMed, MEDLINE, and Embase using PRISMA guidelines, and required ≥ 20 participants who were ≥ 16 years, alongside costs at 1-year postoperatively. The Shapiro–Wilk test, independent sample t-tests, ANOVA, and Spearman’s R were used, with costs adjusted to 2024. A random-effects model was used to compare cost-effectiveness. Bias assessment was according to the Cochrane Risk of Bias 2.0 tool and the Newcastle–Ottawa Scale.

**Results:**

7 studies were included, with a sample of 6493 participants (3418 M, 3075 F). 2932 and 3511 participants underwent CEA and CAS respectively. CEA reported a significantly longer mean length of procedure (191.92 vs. 77.5 min, p < 0.0001) and length of stay (3.13 vs. 2.60 days, p < 0.0001) vs. CAS. The mean adjusted cost of CEA and CAS were $18156.60 (6466) and $17711.01 (5511) respectively. Studies reported lower risks of stroke (2.12% vs. 3.65%, p < 0.001), higher risks of myocardial infarctions (1.70% vs. 1.42%, p < 0.01), and higher risks of other complications for CEA vs. CAS respectively. The expected 1-year cost of CEA was marginally lower than CAS ($21264.03 vs. $21433.14, p < 0.05). The cost-effectiveness of CEA was marginally better than CAS (ratio = 1.019, 95% CI [1.017, 1.020)].

**Conclusions:**

CEA provides marginally improved cost-effectiveness over CAS, providing long-term cost benefits to centers with large surgical volumes. However, shorter procedural times and inpatient stays with CAS may improve overall productivity. Cost should hence not be a deciding factor when choosing between CEA and CAS.

## Introduction

Carotid artery stenosis is a significant risk factor for ischemic stroke, accounting for approximately 10–20% of all incidences [1]. As the burden of stroke continues to rise globally, the management of carotid artery stenosis has become an important focus in preventive medicine. Two primary interventional approaches have emerged as the standard treatments of severe carotid artery stenosis: carotid endarterectomy (CEA) and carotid artery stenting (CAS) [2].

Carotid endarterectomy has long been considered the gold standard for treating severe carotid artery stenosis. The procedure involves removing atherosclerotic plaque from the affected artery, thus restoring blood flow to the brain [3]. Contrastingly, carotid artery stenting offers a minimally invasive alternative, involving the endovascular placement of a stent to widen the narrowed artery lumen to restore cerebral perfusion [4].

Despite both procedures aiming to reduce the risk of stroke, they differ in their associated risks, approaches, complication profile and indications. Several studies have compared the efficacy and safety of CEA and CAS. Some studies suggest comparable outcomes while others indicate differences in operative risk and long-term outcomes [5].

However, economic implications of these procedures cannot be overlooked and is a crucial consideration for healthcare systems and clinicians. Factors such as procedural costs, length of hospital stay, rates of complications, and long-term outcomes all contribute to the overall economic efficiency of these interventions. Given that both CEA and CAS generally offer good long-term efficacy in preventing strokes, cost-effectiveness becomes a significant factor that helps health systems divide between the two procedures.

Despite the importance of this topic, there is paucity of comprehensive analyses which synthesize the current literature on cost-effectiveness of CEA compared to CAS [6]. Cost-effectiveness analysis is a crucial tool in healthcare decision-making, helping health systems determine which treatments provide the best value for money. The most commonly used metric in cost-effectiveness studies is the quality-adjusted life year (QALY), which combines both the quantity and quality of life gained from an intervention. This systematic review and meta-analysis aims to address this gap by evaluating and comparing the cost-effectiveness of CEA and CAS for the management of carotid artery stenosis. The findings of this study seek to not only inform clinical decision-making but also contribute insights for resource allocation in healthcare and the management of carotid artery stenosis.

## Methods

### Structured Search

A structured search was conducted in accordance with guidelines outlined by the Preferred Reporting Items for Systematic Review and Meta-Analysis (PRISMA) checklist [7]. Articles were searched using PubMed, MEDLINE, and Embase from conception to July 2024. No restrictions or filters were used for date of publication or study design. Only articles in English were included. The keywords “(Carotid Endarterectomy OR Carotid Surgery OR Carotid Plaque Surgery OR Carotid Stenosis Surgery OR Carotid Revascularization OR CEA) AND (Carotid Stenting OR Carotid Angioplasty OR Endovascular Carotid Surgery OR Carotid Stenosis Stenting OR CAS) AND (Cost OR Cost-Effectiveness OR QALY)” were used in MeSH and text-word format to retrieve journal articles.

### Inclusion and Exclusion Criteria

Inclusion was restricted to double-intervention clinical trials and cohort studies that were published in English. Articles reporting only simulated data were excluded, but studies with original sample interventional groups using generalised data for the risk of complications were included. Studies were required to have a sample size of ≥ 20 participants, all of whom were ≥ 16 years of age. No restriction was placed on the etiology or severity of stenosis, although this was recorded based on studies. Included studies were required to measure the cost, general risk, or cost-effectiveness of both procedures at 1 year following intervention.

### Data Extraction

Studies from the database search were input into the systematic review software *Covidence* (Veritas Health Innovation, Melbourne, Australia). Three reviewers independently completed initial abstract and title screening, followed by full-text screening in accordance with the inclusion and exclusion criteria. Any conflicts in decisions were discussed until a consensus was reached with the assistance of a senior author. The following study characteristics and patient variables were extracted:

*Author, study name, study design, sample size (for both groups), distribution of sex, population age (mean, median, range), etiology of stenosis, severity of stenosis, length of procedure, length of stay, the base cost of the procedure, risks of complications contributing to further costs within 1 year, cost of complications, and cost per QALY*.

### Statistical Analysis

Independent-sample t-tests were performed to compare demographic, treatment, and cost characteristics across both treatment groups. ANOVA tests were performed to directly compare the risk of stroke, myocardial infarctions (MI), and other complications across both treatment groups in the studies reporting them. For studies reporting cost-values in currencies other than USD, cost-values were calculated based on the exchange rate during the month and year at which the data was collected based on the national bank conversion rates from which the study originated. Cost was adjusted for inflation using the United States Bureau of Labor Statistics calculator [8]. Expected costs of CEA and CAS were calculated using the sum of the cost of the procedure itself, alongside the reported mean costs of complications, multiplied by their respective risk profiles, deriving a comprehensive estimate. The Shapiro–Wilk Test and Spearman’s R were used for correlation-based analyses. The cost-effectiveness of CEA (in QALY/$100,00) vs. CAS (in QALY/$100,00) was used to obtain the ratio of cost-effectiveness per study, with a random-effects model used to assign weightage to derive the pooled estimate of cost-effectiveness.

### Quality Assessment

Quality assessment was conducted independently by all reviewers, with any conflicts discussed with a senior author until consensus was reached. The Newcastle–Ottawa Scale (NOS) was used for observational and cohort studies. The Cochrane Risk of Bias 2.0 tool was used for trials.

### Study Registration

This study was registered with the International Prospective Register of Systematic Reviews (PROSPERO). Registration ID: CRD42024596145.

## Results

**Fig. 1:**
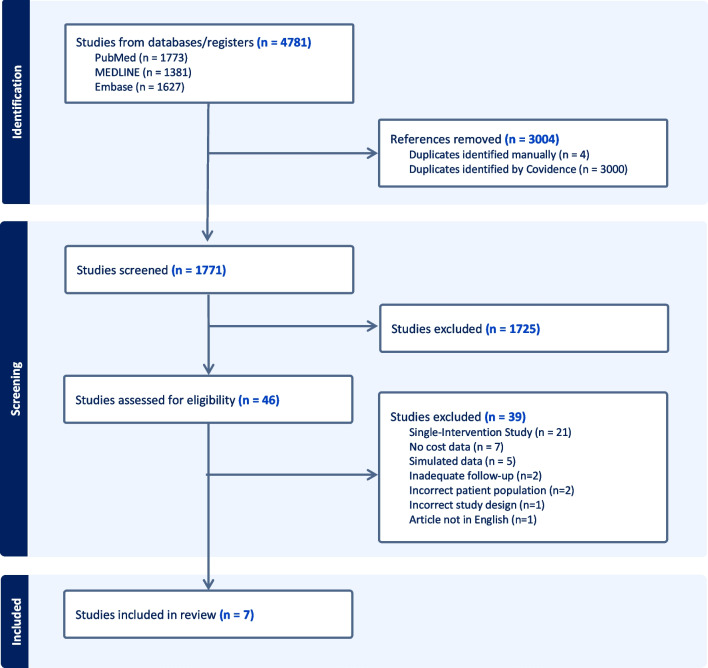
PRISMA diagram of included studies

In all, 4781 studies were obtained during the initial search across all databases, of which 3004 duplicate studies were removed automatically and 4 were removed manually. The remaining 1771 studies underwent title and abstract screening. Following screening, 1725 studies were excluded due to being irrelevant to the investigation, leaving 46 studies that were assessed for full-text eligibility. Of the 46 studies, 39 studies were excluded, primarily due to a lack of both a CEA and CAS group within the sample. This left 7 studies that remained within the final analysis, of which 3 were RCTs, and 4 were cohort studies, of which 3 used a combination of original samples and generalized risk data (Fig. 1).

The total sample across all included studies comprised 6493 participants, of which 3418 were men and 3075 were women. Within the entire sample, 2982 participants underwent CEA, while 3511 underwent CAS. A further subgroup of the analysis, spanning studies directly reporting on the cost effectiveness (in cost per QALY), comprised 4975 participants (2509 CEA, 2466 CAS). While all outcomes reported within this study are up to 1 year following procedural intervention, the mean follow-up for both the CEA and CAS groups was 5.89 years across studies, used to better approximate the general risk of complications and lifetime costs. These findings are described in Table 1.

**Table 1:** Demographic characteristics of CEA and CAS groups in included studies

Demographics		Total (n = 6493)	CEA (n = 2982)	CAS (n = 3511)
Sample Size	Men	3418	1681	1737
	Women	3075	1301	1774
Age (mean, SD)		70.84 (9.3)	71.12 (8.9)	70.60 (9.5)
Length of Procedure (mean, minutes)		130.05 (42)	191.92 (57)	77.5 (33)
Length of Stay (mean, days)		2.84 (1.3)	3.13 (1.4)	2.60 (1.2)
Etiology of Stenosis	Atherosclerotic	6184 (95.20%)	2818 (94.50%)	3366 (95.87%)
	Fibromuscular dysplasia	59 (0.91%)	38 (1.27%)	21 (0.60%)
	Vasculitis	31 (0.48%)	11 (0.37%)	20 (0.57%)
	Radiation Induced	35 (0.54%)	24 (0.80%)	11 (0.31%)
	Moya Moya	64 (0.99%)	38 (1.27%)	26 (0.74%)
	Other/Unknown	120 (1.85%)	53 (1.78%)	67 (1.91%)
Follow-up (mean, years)		5.89		

**Fig. 2:**
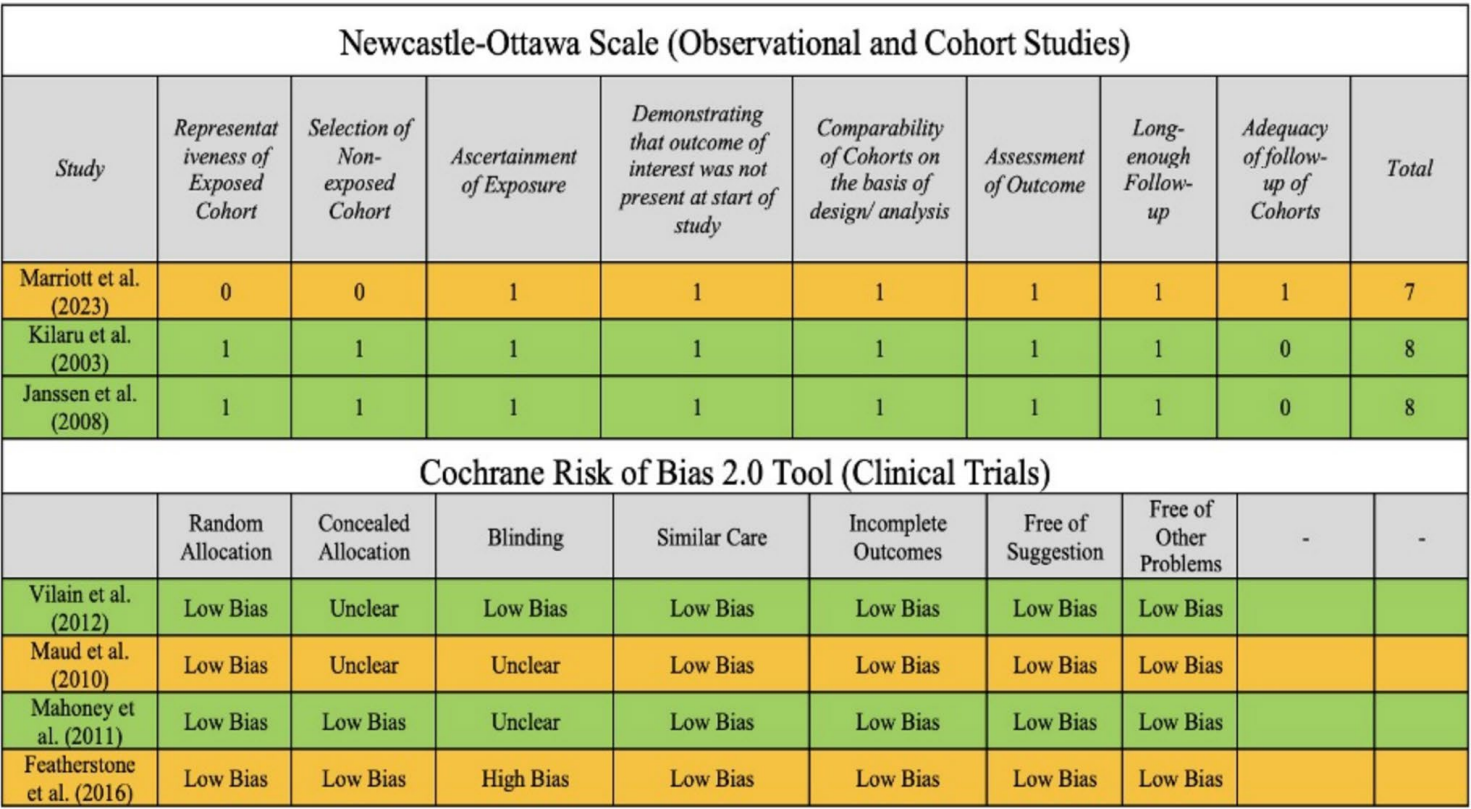
Risk of bias analysis of included studies

3 studies were evaluated using the Newcastle–Ottawa Scale, while 4 were evaluated for bias using the Cochrane Risk of Bias 2.0 tool. All studies scored generally well on bias assessment, with no significant concerns regarding systematic error (Fig. 2). We tested for correlations between study sample size and the reported costs of CEA (r = 0.289, p = 0.64) and CAS (r = 0.747, p = 0.21), complication risks across CEA (r = −0.608, p = 0.15) and CAS (r = −0.06, p = 0.90), and QALY/$100,00 across CEA (r = −0.116, p = 0.85) and CAS (r = −0.03, p = 0.96). We found no evidence that odds values differed between smaller and larger studies in a systematic manner that would indicate publication bias.

In addition, we also performed stratified analyses on complication risks across CEA and CAS for the two studies using generalized/modelled data regarding complication profiles of their samples. No significant differences were observed between the studies using generalized complication data for their cohorts (n = 708) and studies using direct data for their cohorts (n = 5875) in complication profiles (r = 0.04, p = 0.84). Moreover, odds of cost-effectiveness (in QALY/$100,000) across CEA and CAS were compared with studies using direct data, finding no significant differences within the samples (p = 0.81).

CEA was found to have a significantly higher mean length of procedure (minutes) compared to CAS (191.92 vs. 77.5, p < 0.0001), alongside a longer mean length of stay (days) compared to CAS (3.13 vs. 2.60, p < 0.0001). No significant differences were observed in the etiologies or severity of stenosis across both groups (Table 1).

Within the CEA group, comprising a sample of 2982 participants, the mean cost of the procedure itself (including costs of medical equipment, hospital fees, physician fees, and staffing fees) was $12,912.18 (4051). Across all studies (Table 2), when adjusted for inflation, the mean cost of the procedure to the hospital was $18,156.60 (6466). On the other hand, within the CAS group, the mean base cost of the procedure was $12,003.13 (3481), which when adjusted for inflation, was $17,711.01 (5511) in 2024, suggesting a significantly lower cost in the CAS group (p < 0.001).

**Table 2:** Cost and complication risk profiles of CEA and CAS by study

*Carotid Endarterectomy*							
Study ID	Year	Sample	Base Cost (USD)	Base Cost (Adjusted, USD)	Stroke Frequency	MI Frequency	Others Frequency
Marriott et al	2023	150	7272	7504	0.90%	NR	NR
Kilaru et al	2003	447	7871	13454.31	0.90%	1.10%	Hematoma (2.68%), CN Injury (1.78%)
Janssen et al	2008	26	5445	7952.4	6.26%	NR	NR
Vilain et al	2012	1184	14816	20291.8	1.07%	0.25%	NR
Maud et al	2010	167	NR	NR	6%	6%	NR
Mahoney et al	2011	151	19796	27673.42	4%	7.90%	NR
Featherstone et al	2016	857	NR	NR	3.20%	2.10%	NR
*Carotid Stenting*							
Study ID	Year	Sample	Base Cost (USD)	Base Cost (Adjusted, USD)	Stroke Frequency	MI Frequency	Others Frequency
Marriott et al	2023	151	7607	7850.34	0.90%	NR	NR
Kilaru et al	2003	1018	10133	17320.87	5.00%	0.80%	Hematoma (0.8%)
Janssen et al	2008	27	7663	11191.77	6.61%	NR	NR
Vilain et al	2012	1136	15055	20619.14	1.57%	0.17%	NR
Maud et al	2010	167	NR	NR	5%	2%	NR
Mahoney et al	2011	159	7084	9902.94	1%	2.50%	NR
Featherstone et al	2016	853	NR	NR	5.50%	3.50%	NR

The risk of any listed complications was also analysed across both treatment modalities in studies reporting them (Table 2). Within the CEA group, up to 1 year, the expected mean risk of stroke (both major and minor) was significantly lower than the CAS group (2.12% vs. 3.65%, p < 0.001). There was also a marginal but significant difference in frequency of an MI within one year, with the CEA group being more at-risk than the CAS group (1.70% vs. 1.42%, p < 0.01). Some studies reported risks of other complications, namely a risk of 2.68% and 0.8% of hematoma formation in the CEA and CAS groups respectively, alongside a 1.78% risk of CN injury in the CEA group.

Across studies reporting general costs of complications (Table 3), the mean costs of a stroke and MI to the hospital in 1 year were $75550.89 and $66991.71 respectively (adjusted to 2024). The cost of hematoma formation and CN injury were, on average, $2905 and $16238 respectively (adjusted to 2024). Expected costs were computed by the sum of costs of each respective complication scaled to their pooled risk, deriving a pooled estimate of overall cost. Factoring in the risk of complications, CEA was found to have a marginally lower overall 1-year expected cost to the hospital compared to CAS ($21264.03 vs. $21433.14, p < 0.05). As such, at the 1-year mark, CEA would result in savings of $179,110 per 1000 patients to the hospital (inclusive of complications) vs. CAS.

**Table 3:** Expected 1-year costs of CEA and CAS with complication risks

	CEA		CAS	
Cost Component	Cost (Adjusted, USD))	Risk	Cost (Adjusted, USD)	Risk
Procedural	18156.6	1	17711.01	1
Stroke	75550.89	0.0212	75550.89	0.0365
MI	66991.71	0.017	66991.71	0.0142
Hematoma	2905	0.0268	2905	0.008
CN Injury	16238	0.0178	16238	0
Total	21264.03		21443.14	

For studies reporting long-term cost data and estimates (Table 4), CEA was also found to have significantly but slightly reduced overall costs compared to CAS (p < 0.01). The mean 10, 20, and 30-year cost of CEA (including complications and adjusted to 2024) was $166229.31, $311314.89, and $399696.05 respectively. Similarly, the 10, 20, and 30-year costs of CAS was $169494.78, $317247.03, and $406542.08 respectively. The breakdown of long-term costs is described in Table 4.

**Table 4:** Expected long-term costs of CEA and CAS

	CEA			CAS		
Years Since Procedure	Procedural (Adjusted, USD)	Complications (Adjusted, USD)	Total (Adjusted, USD)	Procedural (Adjusted, USD)	Complications (Adjusted, USD)	Total (Adjusted, USD)
10	75333.78	90895.53	166229.31	78808.52	90686.26	169494.78
20	143546.09	167768.80	311314.89	149102.16	168144.87	317247.03
30	187096.42	212599.63	399696.05	192583.08	213959.90	406542.98

**Fig. 3:**
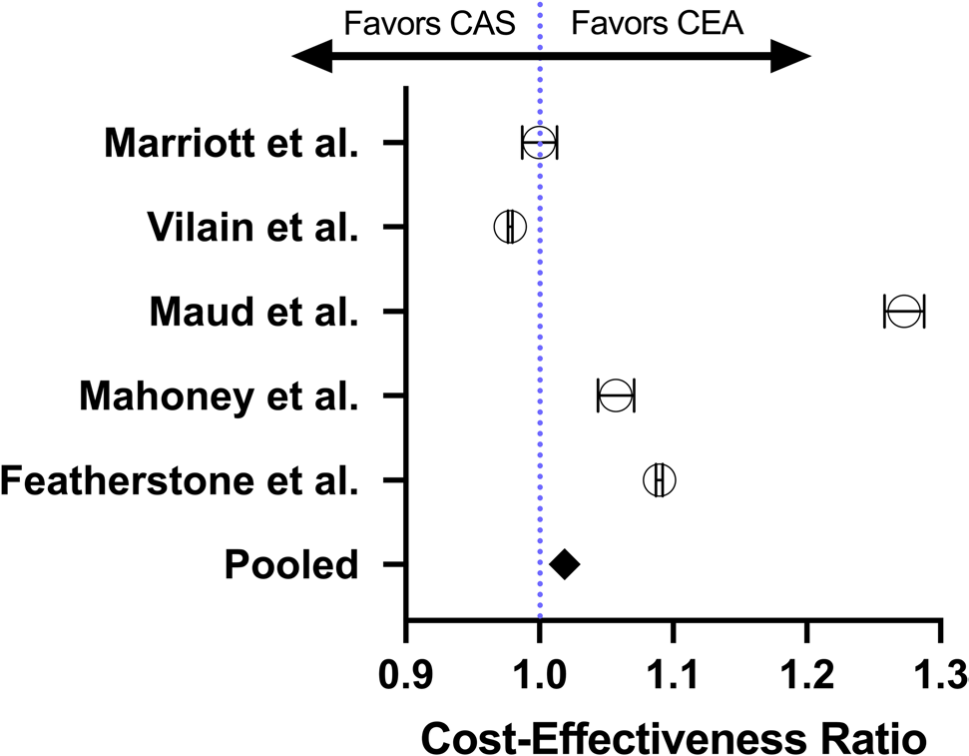
Forest Plot of Odds of Cost-Effectiveness by QALY/$100,000 of CEA vs. CAS by study

Using a random effects model, with the studies directly reporting the cost per QALY of both interventions, a pooled analysis was performed to identify differences in cost effectiveness (n = 4975). All cost-effectiveness values of studies were converted to QALY/$100,000, adjusted to 2024, and compared between CEA and CAS to obtain a ratio. It was found that CEA was significantly more cost-effective than CAS (ratio = 1.019, 95% CI [1.017, 1.020)], but that the difference in the ratio was marginal, offering only slight improvements over CAS. This highlights that for every $100,000 spent towards treating patients with carotid stenosis, CEA would result in savings of approximately 0.019 QALYs for a single patient, with a switch to CEA resulting in 19 QALYs saved per 1000 patients. These findings are described in Fig. 3.

## Discussion

To our knowledge, this is the first study to perform a meta-analysis of the costs and cost-effectiveness of CEA vs. CAS for carotid stenosis. This review presents novel findings, suggesting that CEA is marginally less costly (including the expected cost of complications) and more cost-effective compared to CAS. The study found a cost-effectiveness ratio of 1.019 in favor of CEA, indicating a slight advantage in terms of QALYs gained per $100,000 spent. While the current literature has largely suggested CEA provides a significant benefit in cost-effectiveness due to lower complication risks, the findings of this study suggest that up to 1-year, this increase in cost-effectiveness is sparse [9]. This minimal difference underscores the importance of considering factors beyond cost-effectiveness when making treatment decisions.

In terms of the base cost, across all studies, CAS had a significantly lower cost of the procedure and inpatient stay compared to CEA. However, when adjusted for inflation, this difference was only marginal, with costs only around 2.5% lower than CEA when accounting for staffing, inpatient costs, equipment, and anticoagulation prescribed after both procedures. These findings suggest that when factoring in all costs related to both procedures, CAS does not offer a clinically significant decrease in costs compared to CEA. While still developing, some studies have also recently commented on this discrepancy of the costs of CAS in expectation and reality, with CAS often not offering a significant cost–benefit over CEA, and sometimes even being more expensive procedurally [10, 11].

However, a key aspect of cost is with regards to the risk of complications patients can expect with both procedures, as these can significantly add on to medical charges, creating a complex interaction of factors contributing to the cost-effectiveness of these procedures. While CEA showed a lower risk of stroke within one year compared to CAS (2.12% vs. 3.65%), it was associated with a slightly higher risk of myocardial infarction (1.70% vs. 1.42%). While studies report slight differences in both the risk of complications and the expected cost of each complication to the hospital/patient, our pooled analysis across all studies revealed that CEA emerged as slightly less costly ($21,264.03 vs. $21,433.14) compared to CAS when accounting for complications. This difference is marginal, and suggests that within the follow-up of 1 year following intervention, both procedures can be expected to have approximately equivalent costs within a standard population of patients with carotid stenosis. Nevertheless, while this is relatively insignificant at the individual level, in the context of centers performing a high volume of procedures annually, we found savings of $179,100 per 1000 patients treated, which can, in the long run, provide significant benefit.

For long-term costs, at 10, 20, and 30 years, CEA generally resulted in lower costs to the patient/hospital, but only within margins of 1.5–3%. In our search, long-term cost data for both procedures were highly scarce, with only 2 articles reporting on projections up to 30 years based on follow-up data since intervention, preventing us from establishing significance. While this is expected, especially due to the relative novelty of procedures such as CEA and especially CAS in treating carotid stenosis, it highlights a significant gap in our current knowledge of long-term cost-effectiveness of these procedures [9]. Further prospective data of cohorts of patients undergoing CEA/CAS at longer follow-up intervals is required to better gauge the risk of long-term complications, medications, and costs.

Within the direct analysis of cost-effectiveness based on the QALY/$100,000 per procedure, our analysis revealed a pooled ratio of CEA to CAS of 1.019, with CEA significantly associated with a greater cost-effectiveness. This finding is in line with our calculated procedural and overall cost across studies, reinforcing the significant but marginal improvement in cost-effectiveness that CEA provides over CAS.

An interesting finding of our analysis was that CEA required substantially more time than CAS (191.92 vs. 77.5 min), and patients undergoing CEA had longer hospital stays compared to CAS patients (3.13 vs. 2.60 days). While not directly related to the costs and cost-effectiveness of the procedures, longer procedural durations and turn-around times have been known to significantly improve levels of productivity, especially in high-volume centers performing multiple interventions daily [12, 13]. While our analysis has not explored the differences in overall productivity across the two procedures, it plays a highly relevant role in the ability of clinicians to treat patients more frequently. Further prospective work is required to characterize the potential gains in productivity that CAS can bring, and whether this outweighs the slight cost-effectiveness advantage of CEA.

Our findings reveal a common trend between the procedural cost, overall cost, long-term costs, and cost-effectiveness of CEA and CAS, with CEA generally offering statistically significant but marginal improvements over CAS. On one hand, the consistent gains in cost-effectiveness in CEA may suggest that in cases of ambiguity between the 2 procedures after medical considerations have been made, CEA should be chosen over CAS to optimize the cost to the patient and hospital. This may be particularly relevant to patients with recurrent stenosis and health centers with large volumes of patients treated interventionally for carotid stenosis, for whom the marginal gains in cost can add-up over time to result in significant savings. Studies have highlighted the high concentration of procedures such as CEA being preferentially carried out in high-volume centers, with only 2–10% of patients undergoing intervention in “low-volume” centers [14]. According to a study by Lichtman et al. [15], 231,707 patients were treated for carotid stenosis between 1999–2014 (with this only increasing today) in the United States, with the majority of these patients treated at high volume centers. As such, we estimate cost-savings within the range of $41.5 million USD across 15 years (or $2.76 million USD per year) following the implementation of CEA, which is likely an underestimate considering the lack of inclusion of private procedures and the increased uptake of CAS following 2014. As such, across a large sample of procedures, the reduced costs of CEA can provide substantial benefits to patients and health systems. On the other hand, the findings also suggest that on an individual basis, no one procedure offers a substantial increase in cost-effectiveness and savings than the other. This is especially relevant to patients, the majority of which do not require recurrent procedures in cases of both CEA and CAS, and hence would be unlikely to benefit from the marginal improvements of CEA [16]. As such, while large-volume centers may be incentivized to offer CEA at higher rates based on its higher cost-effectiveness, clinicians should not consider economic factors when deliberating whether CAS should be offered on an individual level, especially if patients would prefer a less-invasive approach.

As ever, nuance is required when considering economic factors between CEA and CAS for carotid stenosis. Clinicians and patients should be aware that cost and cost-effectiveness are not clinically significant differentiating factors when deciding between CEA and CAS at an individual level. Instead, decisions should be based on patient needs, considering all factors such as patient’s overall health status and hence readiness for surgery, surgeon’s expertise, patient preferences concerning invasiveness and recovery duration, and anatomical considerations such as level and degree of stenosis, plaque characteristics, vessel tortuosity, tandem lesions, aortic arch anatomy, and adequacy of collateral circulation [17, 18]. In clinical practice, the cost-effectiveness of carotid endarterectomy (CEA) versus carotid artery stenting (CAS) must be carefully weighed against individual patient differences to optimize outcomes. While CEA is often more cost-effective in the long term, CAS may be preferred in high-risk surgical candidates, such as those with severe comorbidities or unfavorable anatomy for open surgery. Additionally, factors like patient age, degree of stenosis, and risk of embolic events influence the selection of the most cost-effective approach. Ultimately, a personalized, evidence-based strategy is required to ensure that both economic considerations and patient-specific risk profiles are balanced to achieve the best clinical outcomes. This study also provides valuable information to centers undertaking a high volume of procedures, emphasizing the gains in cost-effectiveness that CEA consistently provides. In both cases, clinical decision making requires a multidisciplinary approach to treatment planning, involving neurosurgeons, vascular surgeons, interventional radiologists, and neurologists to ensure optimal outcomes for patients. The minimal difference in cost-effectiveness also suggests that healthcare systems and research groups should continue to invest in training and resources for both procedures to maintain flexibility in treatment options.

This meta-analysis provides a novel and comprehensive comparison on cost-effectiveness between CEA and CAS. It also offers insights into procedural costs, complications rates, and their associated expenses, providing insights to guide decision-making processes. The study based its findings on a large sample size of 6,493 participants, allowing it to establish statistical significance and identify common trends, despite marginal differences in costs. The inclusion criteria also required all studies to have both interventional groups, limiting levels of selection bias that may be experienced with single-intervention studies, while also allowing for costs of procedures and complications to be directly compared. In our data, we also found no significant differences in participant demographics, etiologies of stenosis, or follow-up, suggesting generally comparable cohorts within both treatment groups.

## Limitations

While our study presents interesting findings on the cost-effectiveness of CEA and CAS, there are certain limitations that play a role in the interpretation of the results. Firstly, our study primarily explored cost-effectiveness outcomes up to 1-year following intervention. While this is generally the standard time-interval at which cost and complication data is reported, it prevents us from exploring further long-term data that could be relevant, especially to patients. Factors including increasing risks of complications with age, costs of medication, and recurrence of stenosis can all affect the costs of both procedures, and hence can play a significant role in cost-effectiveness. While 2 studies did provide long-term cost estimations, the relative scarcity of this data in the literature prevented us from establishing significance between the cohorts. Secondly, our inclusion criteria only allowed for articles published in English, potentially leading to the omission of studies that may have provided valuable data and insights from other regions of the world. Similarly, our inclusion criteria required a minimum of 20 patients in the groups per study. While this criterion was added to ensure reasonable sample sizes on which to base calculations of cost, complications, and cost-effectiveness, it may have led to the exclusion of smaller studies, particularly those performed by low-volume centers which may have reported varying cost figures. Moreover, while all studies included real patients, two studies used average/generalized data based on all patients who underwent a procedure at a center (instead of solely those in the sample). The inclusion of generalized data that was not directly derived from the sample of patients (particularly for complication profiles), introduces a degree of bias, as the data may not accurately reflect the outcomes within the group of patients being reported. While these studies only contributed a small fraction of the overall sample of patients (10.9%), and sensitivity analysis/stratified testing revealed no significant bias in their data, it is nevertheless an important consideration when interpreting our pooled complication and average cost estimates. Finally, we had 2 studies (Mahoney et al. and Maud et al.) which reported outcomes of the same randomized control trial (SAPPHIRE), but across different cohorts, leading to differences in cost and complication data. As such, it is possible that due to their data resulting from a similar dataset, there may have been a degree of overlap between the different cohorts used. While it is unlikely that this potential overlap of patients affects our findings, since the sample size of these studies together was 644 patients (9.9% of our overall sample), it is still a potentially relevant factor that could affect the validity of the results from these two studies.

A key limitation of this study is with regards to the heterogeneity in cost structures across different healthcare systems, as costs can vary dramatically between countries and regions. Factors including differences in equipment, wage structures, and healthcare payment systems can all play significant roles in how costs are calculated. In a similar vein, heterogeneity also exists across the baseline patient characteristics and populations across the studies, varying in factors such as age, severity of disease, and socio-economic status. While we tried to correct for heterogeneity by ensuring standardization in currency and inflation, inherent differences in healthcare delivery could not be directly controlled, making direct comparisons difficult and limiting generalizability. Due to the relative novelty of the procedures and the lack of cost-based analyses comparing them, there is currently a lack of studies within the literature with a low degree of heterogeneity within them. We also were unable to match patients across studies based on their baseline clinical characteristics due to a scarcity of sufficient data, preventing us from eliminating potential biases that may have existed across studies. On the other hand, heterogeneity is an inherent part of healthcare, and while it can reduce the comparability of studies, eliminating it altogether would not reflect accurate differences in cost and outcomes in the real world. Thus, while we acknowledge the role of heterogeneity in limiting our ability to make stronger comparisons, we hope it can accurately describe the spectrum of procedural differences across health systems.

To address these limitations, further work in prospective, multi-center studies with standardized protocols would offer beneficial insights into the economics of both procedures. This would help to provide more robust and generalizable data, minimizing the impact of surgeon or hospital variability. Moreover, extending follow-up periods is also critical to offer insights into the long-term cost-effectiveness of these procedures. Finally, conducting subgroup analyses based on healthcare systems and regions could potentially help account for cost structure variation and provide more contextualized findings, improving the generalizability of the study.

## Conclusion

This systematic review and meta-analysis demonstrates that CEA is marginally more cost-effective than CAS for managing carotid artery stenosis. However, the difference is minimal, suggesting that cost should not be a primary factor when choosing between CEA and CAS. Clinicians should instead focus on patient-specific factors, considering the slightly lower stroke risk with CEA against its longer procedural time and hospital stay.

The findings from this study emphasize the need for a personalized approach to patient care, taking into account patient characteristics, preferences, and specific risk factors. The marginal difference in cost-effectiveness also illustrates the importance of maintaining the offering of both procedures within healthcare systems to offer a variety of treatment options. Future prospective studies should aim to further evaluate long-term cost-effectiveness and assess the impact on patient characteristics and system demography on outcomes and cost.

## Data Availability

No datasets were generated or analysed during the current study.

## Declarations

### Statement on Publication

This work is not under consideration for publication elsewhere, with the work approved by all authors. There have been no prior submissions or previous reports, or preprints.
